# Cellular components of tumor microenvironment: understanding their role in lymphatic metastasis of tumors

**DOI:** 10.3389/fphar.2024.1463538

**Published:** 2024-12-11

**Authors:** Ziyi Wang, Zehui Li, Xiangyu Sun, Wanfu Men, Yan Xu

**Affiliations:** ^1^ Department of Surgical Oncology and General Surgery, First Hospital of China Medical University, Shenyang, Liaoning, China; ^2^ Department of Thoracic Surgery, National Cancer Center/National Clinical Research Center for Cancer/Cancer Hospital, Chinese Academy of Medical Sciences and Peking Union Medical College, Beijing, China; ^3^ Department of Thoracic Surgery, First Hospital of China Medical University, Shenyang, Liaoning, China; ^4^ Cancer Hospital of China Medical University, Cancer Hospital of Dalian University of Technology, Liaoning Cancer Hospital and Institute, Shenyang, Liaoning, China

**Keywords:** tumor microenvironment, immune cell, lymph node, lymphatic metastasis, cancer

## Abstract

Metastasis is the leading cause of cancer-related death in cancer patients. Tumor cells primarily spread through the hematogenous and lymphatic system. The underlying mechanisms of hematogenous metastasis have been well described over the past few decades. However, the understanding of the molecular mechanisms involved in lymphatic metastasis is still at an early stage. Tumor microenvironment (TME), primarily consisting of T cells, B cells, tumor-associated macrophages, neutrophils, and cancer-associated fibroblasts, has been implicated in the development of lymphatic metastasis. Recent studies have been reported that the dynamic and complex interplay between these cellular components of TME has great effects on lymphatic metastasis. Here, we discussed the paradoxical roles of these cellular component within the TME during lymphatic metastasis, as well as potential therapeutic opportunities to re-educate these cells within the TME to have anti-tumorigenic effects.

## Introduction

Tumor metastasis is the primary cause of tumor-related death, referring to the dissemination of tumor cells from the primary tumor to organs to initiate tumor outgrowth (Valastyan and Weinberg, 2011). Tumor metastasis primarily depends on the blood and lymphatic system. In recent years, the research on the molecular mechanism of hematogenous metastasis of tumor has been more in-depth. However, the molecular mechanism of lymphatic metastasis is still at an early stage. Growing evidence revealed that lymphatic vessel growth (also named as lymphangiogenesis) is an important prognostic indicator of distant metastasis risk and overall survival in multiple cancer types (Sundar and Ganesan, 2007). Epithelial tumors usually first spread through lymphatic vessels to their draining lymph nodes, and the microenvironment of lymph node metastatic tumor is critical for tumor development and response to treatment (Liu et al., 2022; Chan and Zhang, 2022). Herein, it is necessary to explore the molecular mechanism of lymphatic metastasis in different cancer types, which may lay a theoretical foundation for promising anti-tumor strategies.

It has been reported that the dynamic and complex interplay between the cellular components of tumor microenvironment (TME) has great effects on lymphatic metastasis. TME remodeling could induce macrophages-dependent lymphangiogenesis in breast cancer (Du et al., 2022). In gastric cancer, cysteine-rich intestinal protein-1 reshapes the TME by CCL5-mediated macrophage recruitment to induce lymphangiogenesis and increases lymphatic permeability for lymphatic metastasis (Wu et al., 2023). TME is a complex cellular environment composed by various types of cells, including multiple immune cells and stromal cells, which may play an essential role on lymphatic metastasis (Quail and Joyce, 2013). Single-cell RNA sequencing is a powerful method to dissect the dynamics of different cellular components and their complex interplay in the TME during lymphatic metastasis. Liu et al. conducted single-cell analysis of primary breast tumors and paired lymph node metastases. Compared with paired breast tumors, activated LAMP3+ dendrite cells exhibited higher enrichment in lymph node metastases and strongly interacted with Tregs through CCL17, which may lead to suppressed activity of T cells in lymph node metastases (Liu et al., 2023). A type of PLA2G2A + cancer-associated fibroblasts that was enriched in HER2+ breast cancer and showed high expression levels of genes that can interact with immune cells. Another single-cell RNA sequencing of osteosarcoma and lymph node metastases revealed that SPP1+macrophages and SELENOP + macrophages were enriched in lymph node metastases and located adjacent to osteoblast cells, interacting with CAFs to form a barrier that blocks T cells and provide a favorable microenvironment for tumor growth (Liu et al., 2024). In this review, we discussed the complex role of diverse cellular components of TME in the pathogenesis of lymphatic metastasis in tumors.

## Mechanisms of lymphatic metastasis

The investigations on the lymphatic metastasis have long been limited due to the lack of molecular markers to distinguish lymphatic vessels from blood vessels within and surrounding the primary tumors. In the recent decades, it has been gradually recognized that lymphatic endothelial cells are characterized by markers including lymphatic vessel endothelial receptor 1 (LYVE-1), prospero homeobox protein 1 (PROX1), podoplanin (PDPN), vascular endothelial growth factor receptor 3 (VEGFR3) etc., (Table 1). Malignant tumors release lymphangiogenic growth factors to induce lymphangiogenesis in primary tumors and in draining sentinel lymph nodes to drive lymphatic metastasis (Hartiala and Saarikko, 2016). Lymphangiogenic factors secreted by pre-metastatic tumors, including VEGF-A, VEGF-C and VEGF-D, are absorbed by lymphatic capillaries around the tumor and transported by the collecting lymphatic vessels to the TDLNs, where they act directly on pre-existing lymphatic vessels to induce lymphangiogenesis (Alitalo and Detmar, 2012). The volume of lymphatic vessels draining the tumor and lymph flow increases. Once metastatic tumor cells spread to their draining lymph nodes, they act as a primary source of lymphogenic factors that enhance chromosome remodeling and structure maintenance (SMC), lymphatic vessel rearrangement and lymphangiogenesis to facilitate distant metastasis. It has been well-established that TDLNs are the first sites of metastasis in various solid tumors—including gastric cancer, breast cancer, colorectal cancer etc., (Abdelfatah et al., 2018; Núñez et al., 2020). Tumor cells located in TDLNs may disseminate to distant organs, indicating a necessary requirement to target lymphatic metastasis to prevent distant metastasis in some cancer patients.

**TABLE 1:** Specific markers of lymphangiogenesis.

Molecules	Function	Reference
LYVE-1	The lymphatic receptor for the extracellular matrix mucopolysaccharide hyaluronan	Jackson (2004)
PROX1	A transcription factor for lymphatic endothelial cell differentiation	Wilting et al. (2002)
PDPN	A mucin-type transmembrane glycoprotein specific to the lymphatic system	Bieniasz-Krzywiec et al. (2019)
VEGFR3	A receptor tyrosine kinase that binds with VEGFC for lymphatic proliferation, migration, and survival	Kuonqui et al. (2023)
VEGFA	The ligand of VEGFR2 that regulates lymphatic proliferation, migration, and survival	Claesson-Welsh and Welsh (2013)
VEGFC	The ligand of VEGFR3 that regulates lymphatic proliferation, migration, and survival	Kuonqui et al. (2023)

## Cellular components of tumor microenvironment and lymphatic metastasis

**FIGURE 1:**
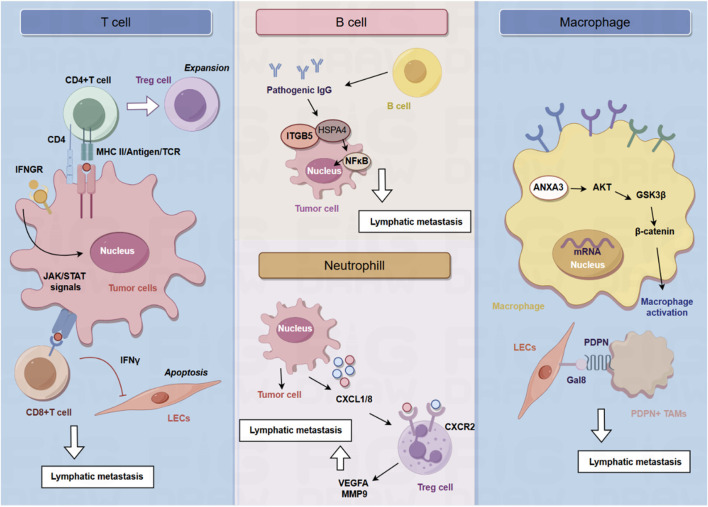
The role of immune cells in the lymphatic metastasis.

**FIGURE 2:**
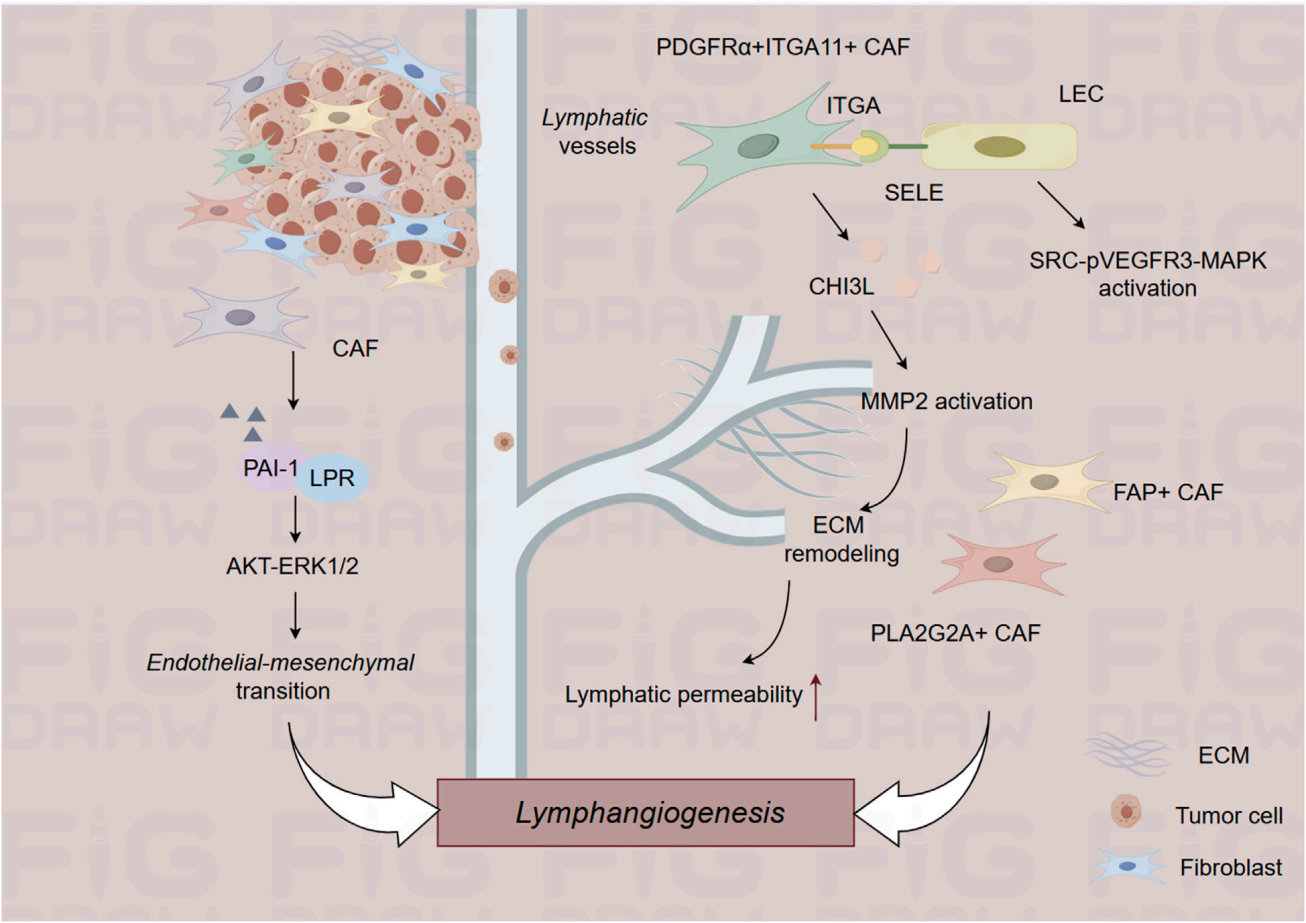
The role of cancer-associated fibroblast in the lymphatic metastasis. CAF, cancer-associated fibroblast; ECM, extracellular matrix.

Given the increased understanding of the critical roles of the TME in tumor initiation and progression, targeting cellular components of the TME has been recognized as a promising therapeutic target for developing anti-tumor strategies. The cellular components of TME are primarily composed of immune cells (Figure 1), including T cells, B cells, tumor-associated macrophages, neutrophils, and cancer-associated fibroblasts (Figure 2). In this section, we summarized current progress in understanding the interplay between cellular components of TME and lymphatic metastasis in different cancer types, highlighting novel opportunities for therapeutic targeting of the TME.

## T cells and lymphatic metastasis

Tumor draining lymph nodes (TDLNs) are essential for the production of tumor antigen-specific T cells and effective anti-tumor immune responses (Schenkel et al., 2021; Huang et al., 2022). Lei et al. found that a subset of breast tumor cells in the TDLNs is characterized by MHC-II upregulation in the absence of co-stimulatory molecules, leading to the expansion of immunosuppressive Treg cells, immune tolerance and evasion of anti-tumor immunity (Lei et al., 2023). MHC-II + tumor cells in the TDLNs induce the generation and activation of Tregs, ultimately leading to the establishment of an immunosuppressive microenvironment in the TDLNs to impair anti-tumor immune responses. Genetic knockout of MHC-II could impair lymphatic metastasis and Treg expansion, and overexpression of the MHC-II trans-activator, Ciita, accelerates lymphatic metastasis (Lei et al., 2023). The effect of cytotoxic T cells on the lymphatic vessels during anti-tumor responses is also important for the process of lymphatic metastasis. Tumoral T cells not only induce immunosuppressive functions of tumoral lymphatic endothelial cells (LECs) but also leads to LEC apoptosis. LEC apoptosis and destruction of lymphatic vasculature by cytotoxic T cells leads to impaired lymphatic flow drainage and metastatic dissemination into lymph nodes. Mechanistically, T cells depend on the Interferon-γ (IFN-γ) signaling to reduce tumoral lymphatic vessels. Reduction of tumoral lymphatic vessels induced by cytotoxic CD8^+^ T cells impair lymph node metastasis in an IFN-γ receptor–dependent manner. Once LECs are lack of the expression of IFN-γ, LEC apoptosis is inhibited, suggesting that IFN-γ is critical for decreasing lymphatic vessel (Garnier et al., 2022).

In human cancer, lymphangiogenic growth factor VEGF-C expression is correlated with lymphatic metastasis and shorten survival, while tumoral expression of VEGF-C promotes lymphatic metastasis and blocking VEGFR-3 signaling could inhibit lymphatic metastasis (Chen et al., 2020; Kong et al., 2020; Paillasse et al., 2022). Lund et al. found VEGF-C functions as a pro-tumor immune-modulatory factor and plays an immunosuppressive role for LECs to scavenge and cross-present antigens for inhibition of cytotoxic T cells. VEGF-C-associated lymphangiogenesis impairs anti-tumor immunity, which further promotes metastasis. In B16 F10 melanomas expressing foreign antigens (OVA), VEGF-C protects melanoma from pre-existing anti-tumor immunity and promotes loss of OVA-specific CD8 (+) T cells. Transfer of naive OVA-specific CD8 (+) T cells leads to dysfunctional activation and apoptosis in murine models. LECs cross-present OVA in TDLNs, and immature LECs clear cross-present OVA. Cross-presented LECs drive proliferation and apoptosis of OVA-specific CD8 (+) T cells. In B16 melanoma, VEGF-C-mediated activation of LECs can cross-present tumor antigens, resulting in dysfunction of CD8^+^ T cells (Lund et al., 2012).

## B cell and lymphatic metastasis

B cells are responsible for the production of immunoglobulin and antibodies (Spencer et al., 2022). As one of the primary cellular components of lymph nodes, B cells are critical for lymphangiogenesis via lymphangiogenic growth factors, indicating that B cells play an essential role in lymphatic metastasis. Gu et al. found that B cells promote the invasive potential of tumor cells to facilitate lymphatic metastasis via producing pathogenic IgG. In a murine model of spontaneous lymphatic metastasis of breast cancer, primary breast tumor mediates the accumulation of B cells in the TDLNs, and B cells promote lymphatic metastasis by producing pathogenic IgG that targets glycosylated membrane protein HSPA4 and activates the HSPA4-binding protein ITGB5 in tumor cells and the downstream Src/NF-κB pathway to achieve CXCR4/SDF1α-induced lymphatic metastasis. The critical role of tumor-educated B cells and pathogenic IgG in the niche formation during lymph node metastasis provides potential targets for anti-tumor strategy (Gu et al., 2019).

## Tumor-associated macrophages and lymphatic metastasis

Macrophages are the most abundant phagocytes in the TME and are often correlated with poor prognosis and therapy resistance (Wang et al., 2021). However, the TME can reprogram the function and activation of macrophages to form tumor-associated macrophages (TAMs). M1 macrophages (Classically activation) are activated by pro-inflammatory factors and regulate anti-tumor responses, while M2 macrophages (Alternating activation) are induced by cytokines including IL-4, IL-13, IL-10. TAMs are typically a subtype of M2 and act as a pro-tumor role (Li et al., 2021). Recent studies have gradually recognized the important role of TAMs in lymphatic metastasis.

The distribution of sinus macrophages across the lymph nodes is not uniform, and they can be subdivided into two categories according to their anatomical location in the lymph nodes: subcapsular sinus macrophages and medullary sinus macrophages. Subcapsular sinus macrophages are the first layer of immune cells exposed to metastatic tumor cells and tumor-derived antigens from afferent lymphatic vessels. It has been found that subcapsular sinus macrophages promote lymphatic metastasis of melanoma via the IL1α-STAT3 axis. Elimination of macrophages in lymph nodes or the administration of an IL1α-specific blocking antibody could reduce metastatic spread (Virgilio et al., 2022).

A large number of VEGFR3-expressing macrophages home in tumors after chemotherapy. Macrophages facilitate lymphangiogenesis and lymphatic metastasis via the VEGF-C/VEGFR3 axis. VEGFR3-expressing macrophages promote lymphangiogenesis mainly via the production of cathepsin, resulting in increased heparinase activity. Therefore, inhibition of the VEGF-C/VEGFR3 pathway could inhibits the activity of chemotherapy-induced macrophages, resulting in reduced lymphangiogenesis in treated tumors (Alishekevitz et al., 2016). Besides, TAMs expressing VEGF-C has been found to reduce the lung metastasis of breast tumor cells, while increasing lymphatic metastasis. These TAMs are characterized by the expression of podoplanin (PDPN), which could interact with normalized tumor blood vessels expressing VEGFR3. Indicating the paradoxical role of VEGF-C-expressing TAMs in redirecting tumor cells to preferentially spread to the lymph nodes rather than to lung, in part by normalizing tumor blood vessels and inducing lymphangiogenesis (Banerjee et al., 2023). Bieniasz-Krzywiec et al. discovered that PDPN is specifically upregulated in a subgroup of TAMs and is involved in the attachment of this subgroup of TAMs to LECs. PDPN binds to LEC-derived lectin 8 (GAL8) in a glycosylation-dependent manner to activate integrin β1. PDPN-expressing macrophages (PoEMs) stimulate local matrix remodeling, promoting lymphangiogenesis and lymphoinvasion. Herein, blockade of integrin β1, macrophage-specific PDPN knockdown or GAL8 inhibition can impair the adhesion of TAM to LECs, inhibit lymphangiogenesis and reduce lymphatic metastasis (Bieniasz-Krzywiec et al., 2019).

A long noncoding RNA, named as Lymph Node Metastasis Associated Transcript 1 (LNMAT1), has been found to be significantly upregulated in LN-positive bladder tumors and correlated with LN metastasis. LNMAT1 has been found to drive tumor-related lymphangiogenesis and lymphatic metastasis by recruiting macrophages. Mechanistically, LNMAT1 epigenetically activates CCL2 expression by recruiting hnRNPL to CCL2 promoter, which leads to increased H3K4 tri-methylation that ensures hnRNPL binding and enhances transcription. Furthermore, LNMAT1-induced upregulation of CCL2 recruits macrophages into the tumor, which promotes lymphatic metastasis via VEGF-C excretion (Chen et al., 2018). Another lncRNA, termed as lymph node metastasis associated suppressor (LNMAS), is downregulated in LN-positive cervical cancer patients and correlated with LN metastasis. LNMAS exerts its anti-LN metastasis effect by competitively interacting with HMGB1 and abrogating the chromatin accessibility of TWIST1 and STC1 to block epithelial-mesenchymal transition and STC1-dependent immune escape from macrophage phagocytosis.

Proteomic analysis of TAMs isolated from laryngeal squamous cell carcinoma (LSCC) tissue from patients with lymphatic metastasis demonstrated that annexin A3 (ANXA3) was upregulated. ANXA3 promotes the polarization of macrophages into an M2-like phenotype via the AKT-GSK3β-β-catenin pathway. ANXA3-rich exosomes from TAMs could inhibit ferroptosis in LSCC cells via the ATF2-CHAC1 axis. Mechanically, ANXA3-rich exosomes inhibit ubiquitination of ATF2 to upregulate the expression of CHAC1, thereby inhibiting ferroptosis in LSCC cells to promote lymphatic metastasis (Xu et al., 2024). Integrin-mediated adhesion of macrophages plays a key role in lymphatic dissemination of breast cancer. TNBC cells induce mRNA alterations in macrophage, leading to β4 integrin-dependent adherence to lymphatic vessels. β4 integrin retain macrophages surrounding LECs, and the release of TGF-β1 drives LEC contraction via RhoA activation. TGF-β1 drives the aggregation of β4 integrins in the plasma membrane of macrophages and further promotes the adhesion of macrophages, indicating the dual function of TGF-β1 signaling in this context (Evans et al., 2019).

## Neutrophils and lymphatic metastasis

Tumor-associated neutrophils (TANs) have emerged as a critical cellular component of the TME. It has been found that TANs infiltration is significantly elevated in lymph node-metastatic bladder cancer and is correlated with poor prognosis. Neutrophil depletion could impair lymphangiogenesis and popliteal lymphatic metastasis. Mechanistically, transcription factor ETV4 enhances bladder cancer cells-derived CXCL1/8 to recruit TANs, increasing VEGFA and MMP9 secretion from TANs to promote lymphangiogenesis and lymphatic metastasis of bladder cancer. Therefore, ETV4 is a therapeutic target of TANs-mediated lymphangiogenesis and lymphatic metastasis of bladder cancer (Zhang et al., 2023). Neutrophil activation also plays a key role in lymphatic metastasis of gastric cancer. Qian et al. found that the expression level of neutrophils polarization-correlated genes (LCN2 and HMGB1) decreases during the process of lymphatic metastasis, while the expression of these genes in the primary tumors is unchanged. It is suggested that neutrophil N2 polarization plays a critical role in lymphatic metastasis of gastric cancer (Qian et al., 2022).

## Cancer-associated fibroblasts and lymphatic metastasis

Cancer-associated fibroblasts (CAFs) are highly versatile cells that actively participate in tumor initiation and development through complex interplay with other cellular components in the TME (Chen et al., 2021). CAFs are composed of diverse subpopulations with distinct phenotypes and functions (Lavie et al., 2022). Recent advances in single-cell RNA sequencing technology have enabled detailed characterization of the complexity and heterogeneity of CAF subpopulations across various cancer types. Zheng et al. depends on single-cell RNA sequencing and spatial transcriptomics to characterize the role of PDGFRα+ITGA11+ CAFs in the lymphatic metastasis of bladder cancer. PDGFRα+ITGA11+ CAFs are correlated with lympho-vascular invasion in bladder cancer, and these CAFs promote lympho-vascular invasion and lymphatic metastasis in bladder cancer. Mechanistically, PDGFRα+ITGA11+ CAFs activate SRC-p-VEGFR3-MAPK pathway to promote lymphangiogenesis by recognizing ITGA11 surface receptor SELE on LECs. Further, CHI3L1 from PDGFRα+ITGA11+ CAFs aligns the surrounding matrix to promote tumor cell intravasation, promoting lympho-vascular invasion and lymphatic metastasis of bladder cancer (Zheng et al., 2024). Another single-cell sequencing of primary tumors and paired lymph node metastasized tumors of breast cancer conducted by Liu et al. found that a subtype of PLA2G2A + CAFs are enriched in HER2+ breast cancer patients that promotes immune infiltration (Liu et al., 2022). In esophageal squamous cell cancer, FAP + CAFs are strongly correlated with lymphatic metastasis (Kashima et al., 2019). These studies illustrated the context-dependent roles of different CAF populations in lymphatic metastasis of malignant tumors.

During the initiation and progression of tumors, tumor cells secrete several factors to recruit CAFs, while CAFs secrete growth factors, inflammatory factors and chemokines, to reconstruct TME and foster lymphatic metastasis of tumor cells. In addition, tumor-derived extracellular vesicles (EVs) are also critical for the interaction between tumor cells and CAFs, leading to lymphatic metastasis of tumors. Li et al. identified LINC00665 (a CAF-related long non-coding RNA) functions as a critical regulator involved in the lymphatic metastasis of bladder cancer. Mechanistically, LINC00665 induces lymphatic metastasis via RAB27B-HGF-c-Myc loop between bladder cancer cells and CAFs. LINC00665 upregulates RAB27B expression and induces H3K4me3 modification on the promoter of RAB27B. Furthermore, RAB27B-mediated EV secretion activates fibroblasts to CAF phenotype, which reciprocally induces LINC00665 overexpression to form a RAB27B-HGF-c-Myc positive feedback loop, promoting lymphangiogenesis and lymphatic metastasis of bladder cancer. Blockade of EV-transmitted LINC00665 could abrogate lymphangiogenesis of bladder cancer *in vivo* (Li et al., 2023). CAF-secreted PAI-1 induces endothelial-mesenchymal transition (EndoMT) of lymphatic endothelial cells in cervical squamous cell carcinoma. Lymphatic endothelial cells undergoing EndoMT could initiate tumor lymphangiogenesis that promote intravasation and extravasation of tumor cells. Mechanistically, PAI-1 activates the AKT/ERK1/2 pathways by directly interacting with low-density lipoprotein receptor-related protein (LRP1), resulting in enhanced EndoMT process in lymphatic endothelial cells. Targeting CAF-secreted PAI-1 could impair EndoMT and ultimately block CAF-mediated tumor lymphangiogenesis (Wei et al., 2023).

## Conclusion and perspectives

It has been well-established that lymph node status has great influence on the prognosis of tumor patients. Moreover, lymphangiogenesis and lymphatic metastasis are important prognostic markers of metastasis risk and overall survival. Mechanistically, lymphatic metastasis is the result of the cooperative effects of tumor cells, TME and pre-metastatic niches. Given its potential contribution to tumor metastasis and immune escape, lymphangiogenesis has emerged as an attractive therapeutic target for developing novel anti-tumor therapies. Therapies targeting the VEGF-C/VEGF-D pathway with anti-lymphangiogenesis activity have entered clinical trials. The cellular components of TME plays important roles in mediating lymphatic metastasis, which may provide potential targets for novel anti-tumor strategy in the future.
